# Whole-Exome Sequencing Reveals the Genomic Features of the Micropapillary Component in Ground-Glass Opacities

**DOI:** 10.3390/cancers14174165

**Published:** 2022-08-27

**Authors:** Fanchen Meng, Yi Zhang, Siwei Wang, Tongyan Liu, Mengting Sun, Hongyu Zhu, Guozhang Dong, Zhijun Xia, Jing You, Xiangru Kong, Jintao Wu, Peng Chen, Fangwei Yuan, Xinyu Yu, Youtao Xu, Lin Xu, Rong Yin

**Affiliations:** 1 Jiangsu Key Laboratory of Molecular and Translational Cancer Research, Department of Thoracic Surgery, Jiangsu Cancer Hospital & Nanjing Medical University Affiliated Cancer Hospital & Jiangsu Institute of Cancer Research, Nanjing 210009, China; 2 Department of Pathology, Jiangsu Cancer Hospital & Nanjing Medical University Affiliated Cancer Hospital & Jiangsu Institute of Cancer Research, Nanjing 210009, China; 3 Department of Science and Technology, Jiangsu Cancer Hospital & Nanjing Medical University Affiliated Cancer Hospital & Jiangsu Institute of Cancer Research, Nanjing 210009, China; 4 Biobank of Lung Cancer, Jiangsu Biobank of Clinical Resources, Nanjing 210009, China; 5 Collaborative Innovation Center for Cancer Personalized Medicine, Nanjing Medical University, Nanjing 211116, China; 6 College of Basic Medicine and Clinical Pharmacy, China Pharmaceutical University, Nanjing 210009, China

**Keywords:** ground-glass opacities (GGOs), lung adenocarcinoma (LUAD), microdissection, micropapillary (MPP), whole exome sequencing (WES)

## Abstract

**Simple Summary:**

The micropapillary component in lung adenocarcinoma has a valid predictive role for patient prognosis. To investigate targeted clinical strategies, we collected 31 stage I lung adenocarcinoma samples and performed microdissection to separate micropapillary and non-micropapillary components, followed by whole exome sequencing. We focused on the genomic features, evolutionary advantages, and associated clinical implications of the micropapillary component, and we proposed and validated the possible presence of essential mutations, TP53 and ZNF469, in the formation of the micropapillary component. These features may be associated with the formation of high-grade invasive patterns in this component.

**Abstract:**

Background: Micropapillary components are observed in a considerable proportion of ground-glass opacities (GGOs) and contribute to the poor prognosis of patients with invasive lung adenocarcinoma (LUAD). However, the underlying mutational processes related to the presence of micropapillary components remain obscure, limiting the development of clinical interventions. Methods: We collected 31 GGOs, which were separated into paired micropapillary and non-micropapillary components using microdissection. Whole-exome sequencing (WES) was performed on the GGO components, and bioinformatics analysis was conducted to reveal the genomic features of the micropapillary component in invasive LUAD. Results: The micropapillary component had more genomic variations, including tumor mutation burden, intratumoral heterogeneity, and copy number variation. We also observed the enrichment of AID/APOBEC mutation signatures and an increased activation of the RTK/Ras, Notch, and Wnt oncogenic pathways within the micropapillary component. A phylogenetic analysis further suggested that ERBB2/3/4, NCOR1/2, TP53, and ZNF469 contributed to the micropapillary component’s progression during the early invasion of LUAD, a finding that was validated in the TCGA cohort. Conclusions: Our results revealed specific mutational characteristics of the micropapillary component of invasive LUAD in an Asian population. These characteristics were associated with the formation of high-grade invasive patterns. These preliminary findings demonstrated the potential of targeting the micropapillary component in patients with early-stage LUAD.

## 1. Introduction

According to the latest statistics, lung cancer ranks first in terms of incidence and mortality among all malignant tumors in China, with lung adenocarcinoma (LUAD) accounting for approximately 60% of all lung cancer cases [1]. With the orderly implementation of extensive lung cancer screening, an increasing number of LUADs are diagnosed early; however, it has been reported that a significant proportion of patients with early-stage lung cancer experience a recurrence or metastasis within 5 years after clinical intervention [2]. At this stage, a combination of different components of lung cancer tissue, such as lepidic, mucinous, acinar, papillary, solid, and micropapillary, is a useful predictor of the prognosis [3]. Among these components, micropapillary (MPP) components express more aggressive molecular markers; moreover, the proportion of MPP components in tumor cells predicts the risk of early metastasis and lymphatic infiltration and is significantly associated with five-year survival [4,5,6,7].

The MPP component is most common in ground-glass opacities (GGOs) and tends to be distributed at the margins of tumor invasion into normal tissue, with an unexpected inside-out growth pattern [8]. The side of the tumor cell facing the stroma has apical secretory properties, and the secretory content includes stromal and vascular invasion factors that promote tumor multiplication and metastasis. These cellular properties largely determine tumor behavior and malignancy.

This component is distributed in some tumors with characteristic mutations; for example, EGFR and BRAF have high mutation frequencies in MPP samples. This component is also enriched for mutations in certain functional pathways such as the PI3K pathway, which is an independent factor in the assessment of tumor recurrence [9,10,11]. Existing MPP-related genomic studies using panel sequencing have proposed that the MPP component also has a stronger genomic instability and higher heterogeneity than other components and contains specific mutated genes due to the increased presence of subclonal mutations in all stages of LUADs. However, the above findings remain fully validated, while a larger somatic mutational profile of the MPP component at the exon level in early-stage LUADs is currently lacking. Thus, our study explored the mutational landscape of the integrated MPP components.

This study aimed to better understand the overall information on mutations in this component and to gain insight into the role of mutational features in early lung adenocarcinoma in the process of cell differentiation and development. We collected a total of 31 GGO samples and performed whole-exome sequencing (WES) of different components, which was the basis to further elaborate the distribution ratios and mutation selection during the development of the MPP component as well as the differences in functional mutations, copy number variants (CNVs), and pathway mutations between tumor components.

## 2. Materials and Methods

### 2.1. Samples and Microdissection

In this study, we used tumor tissue samples from 31 patients with lung malignancies who underwent radical lung cancer surgery at the Affiliated Cancer Hospital of Nanjing Medical University between February 2016 and February 2021 to study the mutational characteristics of tumor tissues. The patients′ postoperative tissue samples were pathologically confirmed to be lung adenocarcinoma-carrying MPP components. According to the eighth edition of the American Joint Committee on Cancer (AJCC)’s cancer staging, all samples collected were stage I. The lung cancer tissue samples were diagnosed by two pathologists, and microdissection was performed after microscopic pinpointing of the MPP component. The MPP component and non-MPP component were isolated from each sample.

In addition, as control data for the genetic variants, we obtained peripheral blood mononuclear cell (PBMC) samples from 31 tumor tissue samples corresponding to patients. The study was approved by the ethics committee of Nanjing Medical University, and informed consent was obtained from the subjects for postoperative sample collection. The clinical information of the above patients is recorded in Supplementary Table S1.

### 2.2. WES

DNA was extracted from the tissue and blood samples using a FastPure FFPE DNA Isolation Kit and a FastPure Blood DNA Isolation Mini Kit V2. The DNA sample concentrations were measured using a Qubit^®^ 3.0 Flurometer (Q33216, Life Technologies, Carlsbad, CA, USA). A DNA small-fragment library was prepared according to the Agilent SureSelectXT Target Enrichment System method and procedure. After the library’s construction, an initial quantification was performed using Qubit 3.0, and the insert size of the library was measured using Agilent 2100. The insert size was determined to be as expected. After the insert size met the expectation, Q-PCR was performed using a Bio-RAD CFX 96 fluorescence quantitative PCR instrument and a Bio-RAD KIT iQ SYBR GRN to accurately quantify the effective concentration of the library (effective library concentration > 10 nM) to ensure the library’s quality. After the libraries passed QC, the raw data were generated in a FASTQ file format by running the double-end sequencing program (PE150) on the Illumina HiSeq sequencing platform according to the manufacturer′s instructions. The sequencing data quality for this project was assessed using the software FastQC v0.11.7, and the data were then compared with the reference genome using software such as BWA. We performed variant detection using GATK software and SNP and InDel annotation using ANNOVAR software [11,12,13,14,15]. The sequencing information of each sample, including the reads number and average coverage, is recorded in Supplementary Table S2.

### 2.3. Oncogenic Signaling Pathway

Based on the mutated MAF files, the known oncogenic signaling pathways in the TCGA cohorts (Oncogenic Signaling Pathways in The Cancer Genome Atlas) of the overall sample were enriched using OncogenicPathways in the maftools package [16]. Each pathway (RTK-RAS, NOTCH, WNT, PIK3CA, and HIPPO) was plotted using Adobe Illustrator, and we labeled the type, function, and frequency of the mutations in each pathway plotted.

### 2.4. Analysis of the Copy Number Variation

To assess the copy number status of the whole genome based on WES data, we modeled the copy number as an integer using the default parameters for SEQUENZA (R package, version 2.1.2) [17] while calculating the mutation ploidy and cellularity. To infer the clonal composition of each tumor sample, the cancer cell fraction (CCF) of each identified mutation was estimated using PyClone (version 0.13.1) [18], and mutations with a CCF greater than 0.1 were defined as clonal mutations. The rest were defined as subclonal mutations. The output of Sequenza was used as the input for PyClone.

### 2.5. Calculation of the dN/dS Value

The dN/dScv package was used to calculate the dN/dS ratio for each component of each sample (including synonymous mutations) to infer the global mutational selection pressure during tumor development, and we selected samples that fit the dN/dScv calculation model for further analysis.

The dispersion of the allele frequencies of somatic mutations in the whole exome sequencing data for each sample was measured using the mutant-allele tumor heterogeneity (MATH) method [19], and this value was used to measure the intratumor heterogeneity (ITH) of the samples included in this study.

### 2.6. Statistical Analysis

Statistical analyses were performed using R (v4.0.5). For comparisons of the continuous variables between groups, Wilcoxon rank-sum tests were used. For comparisons of the categorical variables paired between groups, McNemar chi-square tests were used. All reported *p* values were two-sided. The differences were considered significant when the *p* value was <0.05. Other figures were generated using the R package ggplot2.

## 3. Results

### 3.1. Study Design

To comprehensively study the mutational landscape of the MPP component and the transcriptomic characteristics of tumor cells in early-stage LUAD, we analyzed intraoperatively resected tumors and the corresponding peripheral blood samples from 31 patients with GGOs. The patients’ information is recorded in Supplementary Table S1. After pathological evaluation, 31 of these samples were microscopically excised and divided into MPP and non-MPP components, and WES was performed on each pathological component, followed by a study of the overall mutation characteristics of each component based on mutated genes, copy numbers, base distributions, functional pathway enrichment, and evolutionary selection. A flowchart of the study design is shown in Supplementary Figure S1A.

To capture the overall mutational landscape of the MPP components in GGOs, the somatic mutational profiles of different components within tumors are presented separately below and used as a basis for comparing differences between groups, followed by an assessment of the developmental characteristics of tumor cells based on mutational information.

### 3.2. Mutational Landscape in the MPP Component of GGOs

**Figure 1:**
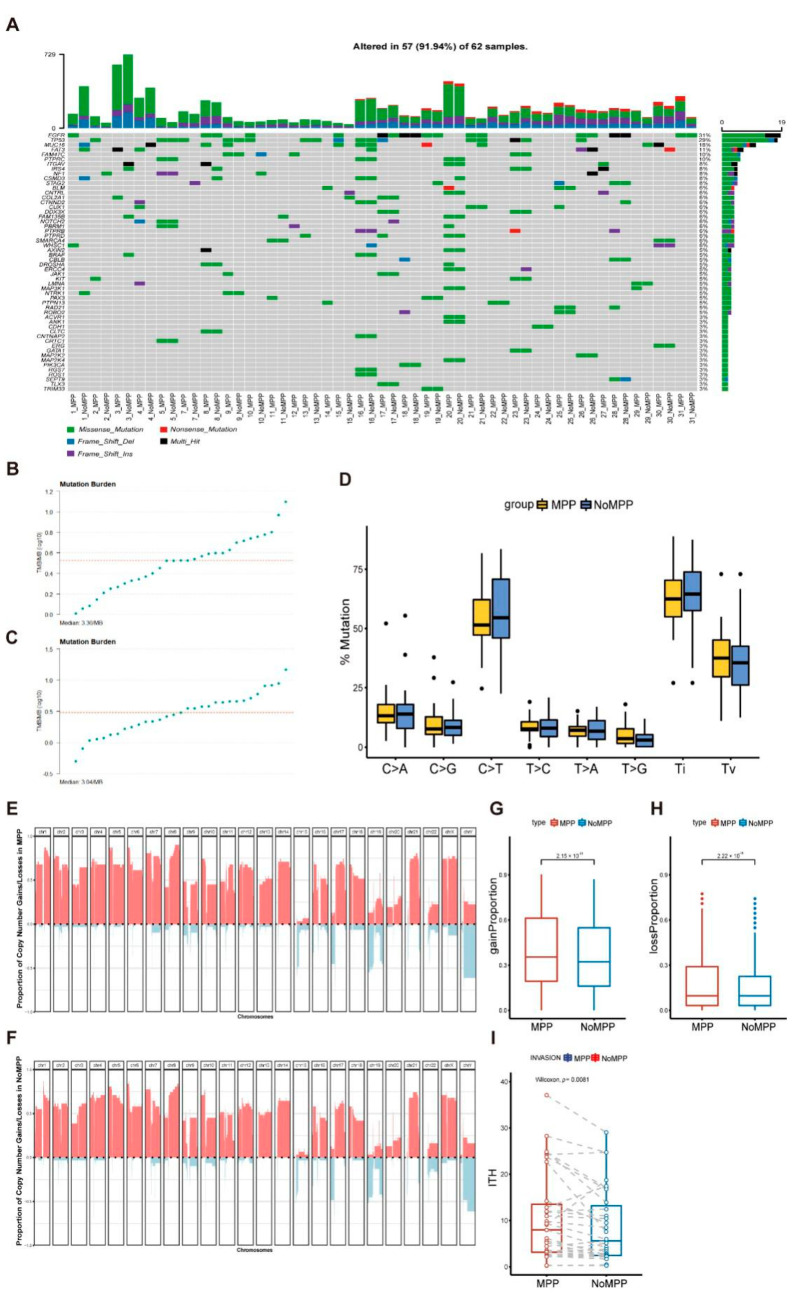
Genetic mutation distribution of the different components in early-stage LUAD. (**A**) Mutation profile of the early-stage LUAD samples divided into micropapillary and non-micropapillary components. (**B**) The tumor mutation burdens (TMB) distribution in a micropapillary component. (**C**) The TMB distribution in a non-micropapillary component. (**D**) The schematics of the base substitution types (C>A, C>G, C>T, T>C, T>A, T>G, Ti, and Tv) in MPP and non-MPP components. (**E**) The copy-number aberration frequency across the MPP of 31 bulk samples. Chromosomes 1–22 and X/Y are positioned along the x-axis, while the y-axis shows the frequency of copy-number gains (pink) and losses (blue). (**F**) Copy-number aberrations frequency across the non-MPP of 31 bulk samples. Chromosomes 1–22 and X/Y are positioned along the x-axis, while the y-axis shows the frequency of copy-number gains (pink) and losses (blue). (**G**) The copy-number aberration (gain) frequency between different components in the same sample. The differences were assessed using a Wilcoxon rank-sum test. (**H**) The copy-number aberration (loss) frequency between different components in the same sample. The differences were assessed using a Wilcoxon rank-sum test. (**I**) Intratumor heterogeneity (ITH) scores of 31 paired MPP and non-MPP components. The differences were assessed using a Wilcoxon rank-sum test.

First, we observed that EGFR and TP53 were the main driver mutations in our study cohort, with most samples showing common driver mutations among different components (MPP and non-MPP) (Figure 1A and Supplementary Table S3). The MPP component shared mutations with the non-MPP component, with higher levels of tumor mutation burden (TMB) (Figure 1B,C). Among the single-nucleotide variants, most were base conversions, with C>T conversions showing a strong predominance in the distribution of substitutions (Figure 1D).

We calculated the CNV for each sample using Sequenza [17]. Heatmaps of the CNV signature distributions for different components in patients P17 and P30 are shown in Supplementary Figure S1B,C, respectively, showing that the different components shared common CNV loci in the same sample, but that the MPP component had a significantly higher CNV load compared with that of the non-MPP component. Therefore, we separately depicted the CNV acquisition and deletion frequency distributions of the MPP and non-MPP components in all samples (Figure 1E,F and Supplementary Table S4). The results were consistent with the features of the single samples. Additionally, the highest variation frequencies of chromosome arm acquisition were distributed at 1q, 8q, and 21q, while chromosome arm deletions were distributed at 15q, 17q, and 19q. To confirm the component dependence of the CNV acquisition and deletion frequencies, we compared these frequencies between the two groups. The results showed significant differences between the groups (Figure 1G,H), suggesting that CNV frequency was associated with the formation of specific functional morphologies in tumor cells.

The intratumoral heterogeneity (ITH) patterns of the paired components in each sample were quantified to estimate the component’s genetic correlations. As expected, the MPP component had higher ITH levels compared with those of the non-MPP component, consistent with the aggressive profile of the MPP component, suggesting an expansionary predominance of this component in early progression [19,20] (Figure 1I), as reported previously [21,22]. These results suggest that, although there is some homology between MPP and non-MPP components, both emerged and retained their respective adaptive mutations during their development, with a more complex genomic landscape in the MPP region relative to the other components.

### 3.3. Analysis of Mutation Signatures and Oncogenic Pathways in the MPP Component of GGOs

**Figure 2:**
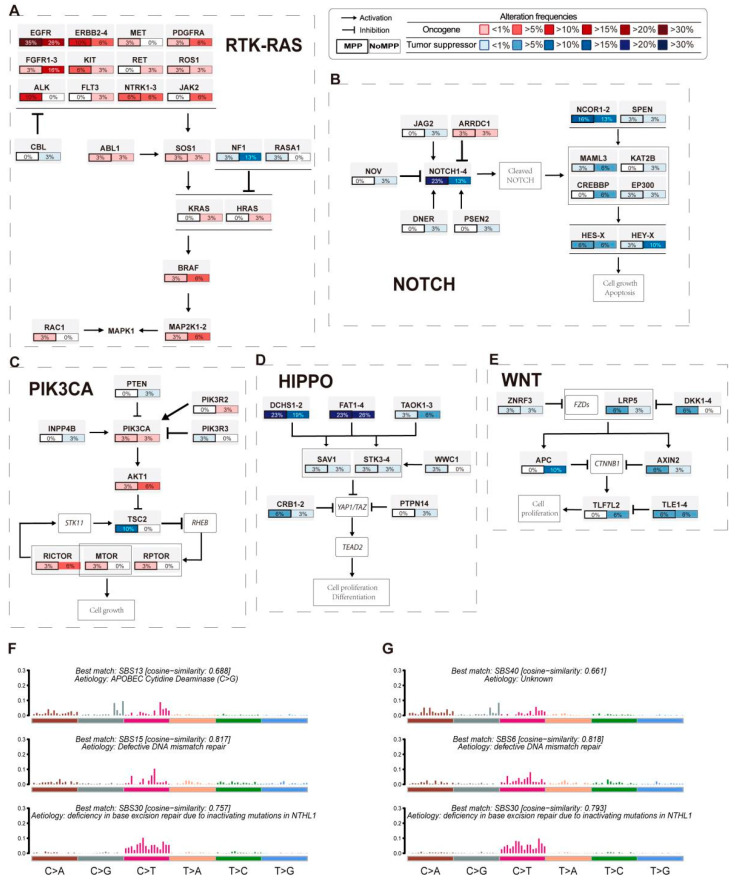
Enrichment analyses of mutation signatures and oncogenic signaling pathways with mutations. (**A**–**E**) Comparison of the alteration frequency of genes in the RTK/RAS (**A**), NOTCH (**B**), PIK3CA (**C**), WNT (**D**), and HIPPO (**E**) pathways between the two components. Mutated genes with oncogenic activation are filled in red, and inactivated tumor suppressor genes are filled in blue. The color intensity represents the frequency of gene replacement occurrence. (**F**) Mutational SBS activities of MPC inferred from base substitution signatures. (**G**) Mutational SBS activities of non-MPC inferred from base substitution signatures.

The MPP component has an apical secretory function and plays an important role in tumor infiltration and angiogenesis [8]. To elucidate the unique biological behavior of the MPP component from the perspective of functional mutations, we analyzed the differences in the distributions of the elements and the requirements of the oncogenic pathway in different components. The RTK/Ras pathway is key to many targetable genes, including EGFR. Integrating the mutations and CNVs revealed that KRAS, ALK, ERBB2-4, and MET were significantly more frequently altered in the MPP component than in the non-MPP component (Figure 2A). This finding was consistent with the previously reported mutational profiles of aggressive tumor components [23]. Thus, mutations in the RTK/Ras pathway are more frequent in the MPP component of GGO patients. In addition, essential mutations related to the Notch and Wnt pathways, including NOTCH1-4, NCOR1-2, LRP5, and DKK1-4, also appeared more frequently in the MPP component (Figure 2B–E). The Notch pathway regulates cell differentiation, feature solidification, cell growth, and apoptosis, while the Wnt pathway plays a role in tumor morphogenesis. Both pathways are associated with the epithelial–mesenchymal transition, which may partly explain the presence of the altered cell polarity in the MPP component.

In addition, the overall base variation layout at the whole-exon level in the MPP component as a whole was more consistent with the SBS13 profile in the Catalogue of Somatic Mutations in Cancer (COSMIC), suggesting an association with the activation of AID/APOBEC cytidine deaminase in cancer [24] (Figure 2F,G), while both fractions exhibited base features associated with DNA mismatch repair defects, including SBS15 and SBS6 [25]. Notably, most MPP components showed alterations in the Notch pathway and were accompanied by an enrichment of mutational features related to the oncogenic activity of AID/APOBEC, similar to the findings reported in other tumors [25,26,27].

### 3.4. Phylogenetic Analysis of GGOs Associated with Mutations in the MPP Component

**Figure 3:**
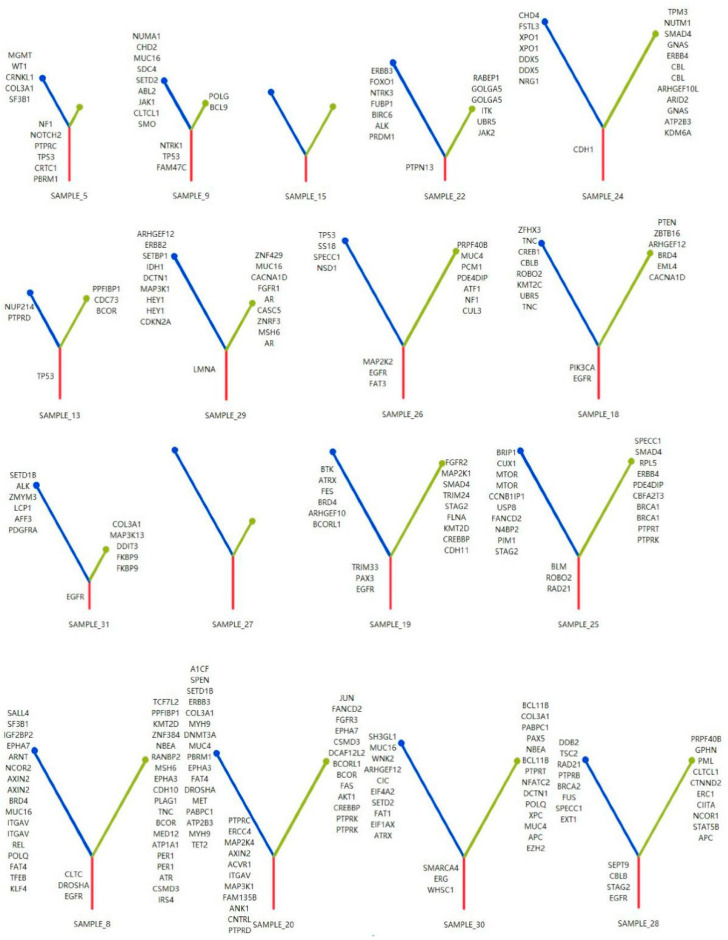
Molecular phylogenetic tree for each GGO lesion. Red, blue, and green lines represent trunk, MPP branch, and non-MPP branch mutations, respectively. The length of molecular time of the trunks and branches was calculated using the number of genomic alterations (mutations and CNVs).

Next, we investigated the genetic changes specific to the MPP component in each patient in this cohort. As shown in Figure 3 and Supplementary Figure S2, we constructed a phylogenetic tree for each patient based on different histological subtypes, with the lengths of the trunk and branches based on the number of mutations, gene-level CNVs, and chromosomal arm-level CNVs [26,27].

Most of the histological subtypes from the same tumor sample source shared multiple genetic events, especially driver mutations, implying homology between the two components as well as pathological subtype differences in the tumor occurring after tumor driver mutations. In addition, the branch length of the MPP component was significantly longer than that of the non-MPP component, suggesting that more genomic alterations may be required for the germination process of the MPP component.

**Figure 4:**
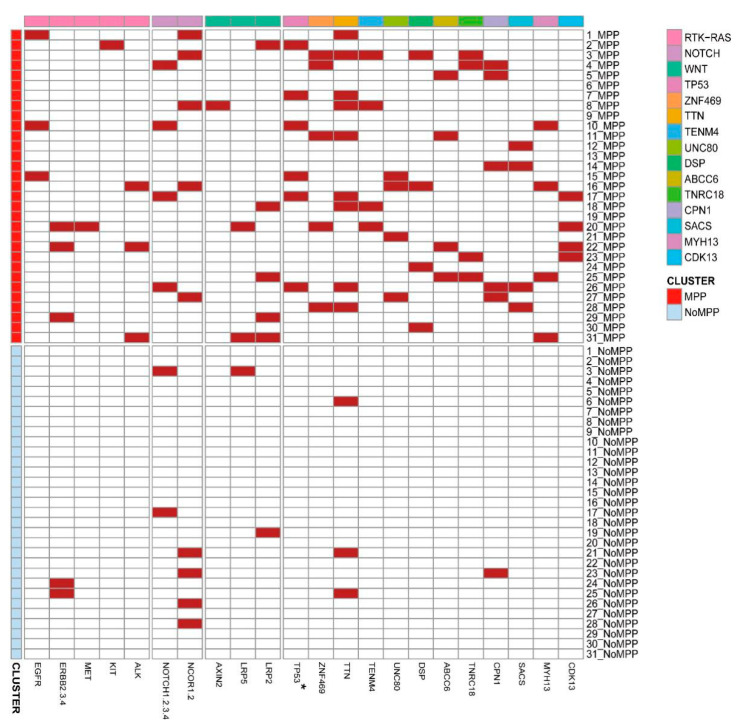
Pathological component-specific mutations for each GGO lesion. Heatmaps were drawn using the distribution of MPP-component branch mutations in the samples after a phylogenetic analysis, and the differences between the two groups were tested using a McNemar chi-square test.

Considering the role of specific mutations in the differentiation of tumor cells toward the MPP component, we counted the frequencies of the distributions of MPP branch mutations without trunk mutations in the samples based on the developmental tree (Figure 4). Within the same sample, genes of the RTK-Ras and Notch pathways were more frequently found in the MPP component. The frequencies of TP53 mutations differed significantly between MPP and non-MPP components (*p* = 0.04), which is a known factor associated with poor prognosis and corresponds to genomic instability features, such as high CNV and TMB, in the MPP component. The gene-encoding zinc finger protein ZNF469 also showed a component-specific distribution (*p* = 0.07).

### 3.5. Distribution of Driver Mutations and Selection Pressure during the Evolution of MPP Components in GGOs

We selected single-nucleotide variants shared between different pathological subtypes of the same sample, which were aggregated with 636 mutated genes localized in all samples, and we further calculated the proportions of the distributions of each mutation using PyClone [18,28]. As expected, the mutated genes in the MPP component were distributed in more tumor cells (Supplementary Figure S1D and Supplementary Table S5) and also had higher frequencies of allelic mutations compared with those of the non-MPP component (Supplementary Figure S1E and Supplementary Table S5). This might be related to the monoclonal population resulting from the stronger proliferative capacity of the MPP component, but it may also be more indicative of the differentiation process of the highly heterogeneous MPP component, with higher genomic variation requirements during differentiation.

dN/dS represents the ratio of non-synonymous to synonymous mutations and is a good means of quantifying the selection pressure during the evolution of the cancer genome. We used the standardized dn/ds algorithm (dN/dScv), filtered out the samples that fit the dN/dScv model, and calculated the ratio of the nucleotide substitution rate at non-synonymous to synonymous sites for each component in the sample to assess the significance of mutational selection pressure on each component during early progression [29,30].

The MPP component showed a significantly higher dN/dS ratio (Supplementary Figure S1F), suggesting that the MPP component is under stronger mutational selection pressure during formation and proliferation. Simply put, to have a chance of survival, this pathological subtype requires a more significant proportion of non-synonymous mutations that result in protein sequence variation or epistatic functional changes.

**Figure 5:**
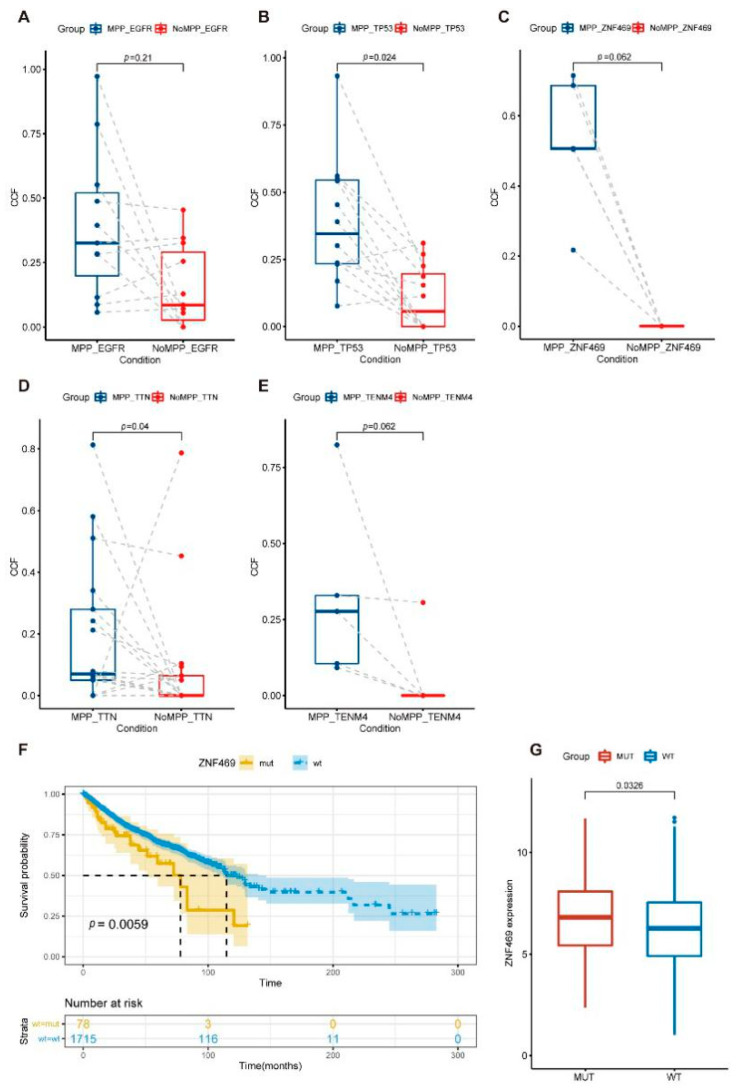
Differential genomic features of MPP components and mutation assessment in pan-cancer. (**A**–**E**) Cancer cell fraction (CCF) of EGFR (**A**), TP53 (**B**), ZNF469 (**C**), TTN (**D**), and TENM4 (**E**) between the MPP and non-MPP components. The differences were assessed using a paired Wilcoxon rank-sum test. (**F**) Survival analysis for ZNF469 mutation in TCGA pan-cancer cohort. (**G**) ZNF469 expression between ZNF469 mutation and wild-type samples in TCGA. The differences were assessed using a Wilcoxon rank-sum test.

The Pyclone results also showed higher abundances of TP53, ZNF469, TTN, and TENM4 mutations in the MPP components (Figure 5A–E). Combining the high-frequency mutations and high tumor cell prevalence mutations occurring in the MPP components, we finally focused on TP53 and ZNF469. TP53 mutations in tumors have been commonly studied. Our analysis of the Cancer Genome Atlas (TCGA) cohort to assess the association of ZNF469 mutations with a prognosis in pan-cancer showed a higher ZNF469 expression and a poorer clinical prognosis in samples with ZNF469 mutations (Figure 5F,G).

## 4. Discussion

Although surgery is the primary route for the clinical treatment of patients with early-stage LUAD, the five-year recurrence rate after this treatment should not be ignored [31,32,33]. Most patients who relapse develop drug resistance and metastasis in subsequent combination therapies and have a poor prognosis. Based on these conditions, the MPP subtype is the most relevant to the malignant behavior of GGO tumors, considering cell differentiation and pathogenesis; moreover, their correlation with clinical prognosis was demonstrated [9]. In the overall structure of the MPP subtype, due to the reversal of cell polarity and the formation of non-structural gaps between tumor cells, these aggregated cell clusters are easily separated and segmented into single cells or smaller cell clusters, which combine with their strong colonization ability to promote invasion and metastasis [8,34,35,36]. Our study compared the genomics of MPP and non-MPP subtypes in an Asian population to investigate the causes of the unique growth patterns of MPP-subtype tumor cells.

We reported the results of a comprehensive genomic analysis of the MPP component in GGOs. This component was initially considered to be homologous to the pathological components and therefore shared common driver mutations and copy number variants at common loci. However, this subtype carries a higher mutational load compared with that of the other components, as evidenced by TMB, CNVs, cancer cell fractions (CCFs), and variant allele frequency. Increasing evidence suggests that tumor cells accumulate mutations during proliferation and invasion and that CNVs at genetic loci play important roles in carcinogenesis and cancer progression [37].

As reported previously, the MPP subtypes tended to show an aggregation of characteristic mutations (EGFR, KRAS, and BRAF) [27,38]; we also observed more frequent mutations in the RTK/Ras, Notch, and Wnt pathways and an enrichment of the AID/APOBEC mutation signature in MPP. Similar to the overall landscape of esophageal, head and neck, and penile squamous cell carcinomas [39,40,41], the MPP showed both enrichment of the Notch pathway mutations and a substitution of the APOBEC-related signature in the presence of high genomic variation. Combined, these findings imply a strong association between these two factors in tumor development.

The phylogenetic tree branches showed that tumor cells require more driver mutations during differentiation into MPP subtypes, which explains the increased mutational selection pressure in this pathological subtype. In addition to the oncogenic signaling pathway genes mentioned in this study, we also observed enrichment trends for TP53, ZNF469, TTN, and TENM4 in the MPP pathological subtypes. We verified that the less-studied ZNF469 was associated with poor tumor prognosis. These results support clinical observations and provide clinical ideas for precise treatment requirements.

Our results demonstrate the concordance between mutational features and cellular functions. This concordance leads us to the next step of our study; that is, whether MPP subtype components with different driver mutations have significant and unique functional characteristics. A correlation between MPP subtypes and immunotherapy efficacy was reported [42], and immunotherapy is closely related to factors such as tumor antigenicity; namely, the MPP composition of the immune microenvironment pair [43] in GGOs corresponds to the tumor microenvironment-related content, a topic that deserves in-depth attention. Our study had the following limitations. First, due to this study cohort′s restricted number and ethnic origin, the genomic characterization of the MPP component needs to be validated in future studies of large multi-ethnic patient cohorts. Second, functional analyses at the transcriptomic and proteomic levels should be continued. Third, a longitudinal analysis at the timescale during tumor formation is still needed to identify the determinants of differentiation in tumors with different components of the same origin. Finally, the heterogeneity of non-MPP subtypes should be considered to reduce the bias in identifying MPP subtypes.

## 5. Conclusions

In conclusion, we reported on the genomic analysis of MPP subtypes. The overall mutational profile of MPP subtypes showed high levels of TMB, CNVs, and CCFs and enrichment for prognosis-related genes such as TP53 and ZNF469. These results provide a solid genomic basis and potential target molecules for the accurate diagnosis and treatment of LUAD.

## Data Availability

All data analyzed during this study are included in this published article and its supplementary information files.

## Supplementary Materials

The following supporting information can be downloaded at: https://www.mdpi.com/article/10.3390/cancers14174165/s1, Supplementary Table S1: Detailed characteristics of the 31 LUAD patients. Supplementary Table S2: DNA sequencing data quality and reference genome comparison statistics. Supplementary Table S3: Detailed somatic mutation data of the 31 LUAD patients classified by cytopathological type. Supplementary Table S4: CNV frequency of the 31 LUAD patients. Supplementary Table S5: Cancer cell fraction and variant allele frequency of the 31 LUAD patients. Supplementary Figure S1: (A) Schematic representation of the analytical strategy. (B,C) Circos plots of the MPC (B) and non-MPC (C) from samples 17 and 30 demonstrate the genome-wide distribution density. The outer ring of the plot shows the chromosomes, and the inner ring exhibits the CNV amplifications (red) and deletions (blue). (D,E) Cancer cell fraction (D) and variant allele frequency (E) of 636 paired trunk mutations between different components. The differences were assessed using a Wilcoxon rank-sum test. (F) dN/dS ratio of paired component-specific mutant genes (exclusive of co-occurring mutated genes) in each sample that were suitable for the dN/dScv model. The differences were assessed using a Wilcoxon rank-sum test. Supplementary Figure S2: Molecular phylogenetic tree of other samples.

## References

[1] Siegel R.L., Miller K.D., Fuchs H.E., Jemal A. (2022). Cancer statistics, 2022. CA Cancer J. Clin..

[2] Jones G.D., Brandt W.S., Shen R., Sanchez-Vega F., Tan K.S., Martin A., Jones D.R. (2021). A Genomic-Pathologic Annotated Risk Model to Predict Recurrence in Early-Stage Lung Adenocarcinoma. JAMA Surg..

[3] Travis W.D., Brambilla E., Nicholson A.G., Yatabe Y., Austin J.H.M., Beasley M.B., Wistuba I. (2015). The 2015 World Health Organization Classification of Lung Tumors: Impact of Genetic, Clinical and Radiologic Advances Since the 2004 Classification. J. Thorac. Oncol..

[4] Chen L., Fan Y., Lang R.G., Guo X.J., Sun Y.L., Cui L.F., Fu L. (2008). Breast carcinoma with micropapillary features: Clinicopathologic study and long-term follow-up of 100 cases. Int. J. Surg. Pathol..

[5] Li W., Han Y., Wang C., Guo X., Shen B., Liu F., Fu L. (2018). Precise pathologic diagnosis and individualized treatment improve the outcomes of invasive micropapillary carcinoma of the breast: A 12-year prospective clinical study. Mod Pathol..

[6] Luna-Moré S., Gonzalez B., Acedo C., Rodrigo I., Luna C. (1994). Invasive micropapillary carcinoma of the breast. A new special type of invasive mammary carcinoma. Pathol. Res. Pract..

[7] Guo X., Chen L., Lang R., Fan Y., Zhang X., Fu L. (2006). Invasive micropapillary carcinoma of the breast: Association of pathologic features with lymph node metastasis. Am. J. Clin. Pathol..

[8] Nassar H. (2004). Carcinomas with micropapillary morphology: Clinical significance and current concepts. Adv. Anat. Pathol..

[9] Zhang Y., Wang R., Cai D., Li Y., Pan Y., Hu H., Chen H. (2014). A comprehensive investigation of molecular features and prognosis of lung adenocarcinoma with micropapillary component. J. Thorac. Oncol..

[10] Warth A., Penzel R., Lindenmaier H., Brandt R., Stenzinger A., Herpel E., Weichert W. (2014). EGFR, KRAS, BRAF and ALK gene alterations in lung adenocarcinomas: Patient outcome, interplay with morphology and immunophenotype. Eur. Respir. J..

[11] Caso R., Sanchez-Vega F., Tan K.S., Mastrogiacomo B., Zhou J., Jones G.D., Jones D.R. (2020). The Underlying Tumor Genomics of Predominant Histologic Subtypes in Lung Adenocarcinoma. J. Thorac. Oncol..

[12] Li H., Durbin R. (2009). Fast and accurate short read alignment with Burrows-Wheeler transform. Bioinformatics.

[13] Kent W.J., Sugnet C.W., Furey T.S., Roskin K.M., Pringle T.H., Zahler A.M., Haussler D. (2002). The human genome browser at UCSC. Genome Res..

[14] Tarasov A., Vilella A.J., Cuppen E., Nijman I.J., Prins P. (2015). Sambamba: Fast processing of NGS alignment formats. Bioinformatics.

[15] Wang K., Li M., Hakonarson H. (2010). ANNOVAR: Functional annotation of genetic variants from high-throughput sequencing data. Nucleic Acids Res..

[16] Mayakonda A., Lin D.C., Assenov Y., Plass C., Koeffler H.P. (2018). Maftools: Efficient and comprehensive analysis of somatic variants in cancer. Genome Res..

[17] Favero F., Joshi T., Marquard A.M., Birkbak N.J., Krzystanek M., Li Q., Eklund A.C. (2015). Sequenza: Allele-specific copy number and mutation profiles from tumor sequencing data. Ann. Oncol..

[18] Roth A., Khattra J., Yap D., Wan A., Laks E., Biele J., Shah S.P. (2014). PyClone: Statistical inference of clonal population structure in cancer. Nat. Methods.

[19] Mroz E.A., Rocco J.W. (2013). MATH, a novel measure of intratumor genetic heterogeneity, is high in poor-outcome classes of head and neck squamous cell carcinoma. Oral. Oncol..

[20] Turajlic S., Sottoriva A., Graham T., Swanton C. (2019). Resolving genetic heterogeneity in cancer. Nat. Rev. Genet..

[21] Shi Q., Shao K., Jia H., Cao B., Li W., Dong S., Fu L. (2022). Genomic alterations and evolution of cell clusters in metastatic invasive micropapillary carcinoma of the breast. Nat. Commun..

[22] Lv J., Shi Q., Han Y., Li W., Liu H., Zhang J., Fu L. (2021). Spatial transcriptomics reveals gene expression characteristics in invasive micropapillary carcinoma of the breast. Cell Death Dis..

[23] Chen J., Yang H., Teo A.S.M., Amer L.B., Sherbaf F.G., Tan C.Q., Zhai W. (2020). Genomic landscape of lung adenocarcinoma in East Asians. Nat. Genet..

[24] Nik-Zainal S., Alexandrov L.B., Wedge D.C., Van Loo P., Greenman C.D., Raine K., Stratton M.R. (2012). Mutational processes molding the genomes of 21 breast cancers. Cell.

[25] Alexandrov L.B., Nik-Zainal S., Wedge D.C., Aparicio S.A., Behjati S., Biankin A.V., Stratton M.R. (2013). Signatures of mutational processes in human cancer. Nature.

[26] Zhang J., Fujimoto J., Zhang J., Wedge D.C., Song X., Zhang J., Futreal P.A. (2014). Intratumor heterogeneity in localized lung adenocarcinomas delineated by multiregion sequencing. Science.

[27] Zhang S., Xu Y., Zhao P., Bao H., Wang X., Liu R., Ma S. (2021). Integrated Analysis of Genomic and Immunological Features in Lung Adenocarcinoma with Micropapillary Component. Front. Oncol..

[28] Zhang C., Zhang L., Xu T., Xue R., Yu L., Zhu Y., Zhang H. (2020). Mapping the spreading routes of lymphatic metastases in human colorectal cancer. Nat. Commun..

[29] Wolfe K., Ó’hUigín C. (2016). Significance of positive selection and gene duplication in adaptive evolution: In memory of Austin L. Hughes. Immunogenetics.

[30] Martincorena I., Raine K.M., Gerstung M., Dawson K.J., Haase K., Van Loo P., Campbell P.J. (2017). Universal Patterns of Selection in Cancer and Somatic Tissues. Cell.

[31] Strauss G.M., Kwiatkowski D.J., Harpole D.H., Lynch T.J., Skarin A.T., Sugarbaker D.J. (1995). Molecular and pathologic markers in stage I non-small-cell carcinoma of the lung. J. Clin. Oncol..

[32] Shedden K., Taylor J.M., Enkemann S.A., Tsao M.S., Yeatman T.J., Gerald W.L., Beer D.G. (2008). Gene expression-based survival prediction in lung adenocarcinoma: A multi-site, blinded validation study. Nat. Med..

[33] Mountain C.F. (2002). Staging classification of lung cancer. A critical evaluation. Clin. Chest Med..

[34] Fidler I.J. (1973). The relationship of embolic homogeneity, number, size and viability to the incidence of experimental metastasis. Eur. J. Cancer.

[35] Liotta L.A., Saidel M.G., Kleinerman J. (1976). The significance of hematogenous tumor cell clumps in the metastatic process. Cancer Res..

[36] Cheung K.J., Ewald A.J. (2016). A collective route to metastasis: Seeding by tumor cell clusters. Science.

[37] Ciriello G., Miller M.L., Aksoy B.A., Senbabaoglu Y., Schultz N., Sander C. (2013). Emerging landscape of oncogenic signatures across human cancers. Nat. Genet..

[38] Kishi N., Ito M., Miyata Y., Kanai A., Handa Y., Tsutani Y., Okada M. (2020). Intense Expression of EGFR L858R Characterizes the Micropapillary Component and L858R Is Associated with the Risk of Recurrence in pN0M0 Lung Adenocarcinoma with the Micropapillary Component. Ann. Surg. Oncol..

[39] Chahoud J., Gleber-Netto F.O., McCormick B.Z., Rao P., Lu X., Guo M., Pettaway C.A. (2021). Whole-exome Sequencing in Penile Squamous Cell Carcinoma Uncovers Novel Prognostic Categorization and Drug Targets Similar to Head and Neck Squamous Cell Carcinoma. Clin. Cancer Res..

[40] Takemoto A., Tanimoto K., Mori S., Inoue J., Fujiwara N., Noda T., Inazawa J. (2021). Integrative genome-wide analyses reveal the transcriptional aberrations in Japanese esophageal squamous cell carcinoma. Cancer Sci..

[41] Sawada G., Niida A., Uchi R., Hirata H., Shimamura T., Suzuki Y., Mimori K. (2016). Genomic Landscape of Esophageal Squamous Cell Carcinoma in a Japanese Population. Gastroenterology.

[42] Gagné A., Enlow W., Pigeon M.A., Orain M., Turcotte S., Bossé Y., Joubert P. (2018). Comprehensive Assessment of PD-L1 Staining Heterogeneity in Pulmonary Adenocarcinomas Using Tissue Microarrays: Impact of the Architecture Pattern and the Number of Cores. Am. J. Surg. Pathol..

[43] Chen Y.P., Lv J.W., Mao Y.P., Li X.M., Li J.Y., Wang Y.Q., Ma J. (2021). Unraveling tumour microenvironment heterogeneity in nasopharyngeal carcinoma identifies biologically distinct immune subtypes predicting prognosis and immunotherapy responses. Mol. Cancer.

