# Supplementary material for: Whole-Exome Sequencing Reveals the Genomic Features of the Micropapillary Component in Ground-Glass Opacities
Source: Cancers (Basel). 2022 Aug 27;14(17):4165. doi: 10.3390/cancers14174165 (PMC9454937; doi:10.3390/cancers14174165)
Supplement: Supplementary file 1 [file cancers-14-04165-s001.zip › cancers-1855002-supplementary-for xml/Fig.S2.pdf]

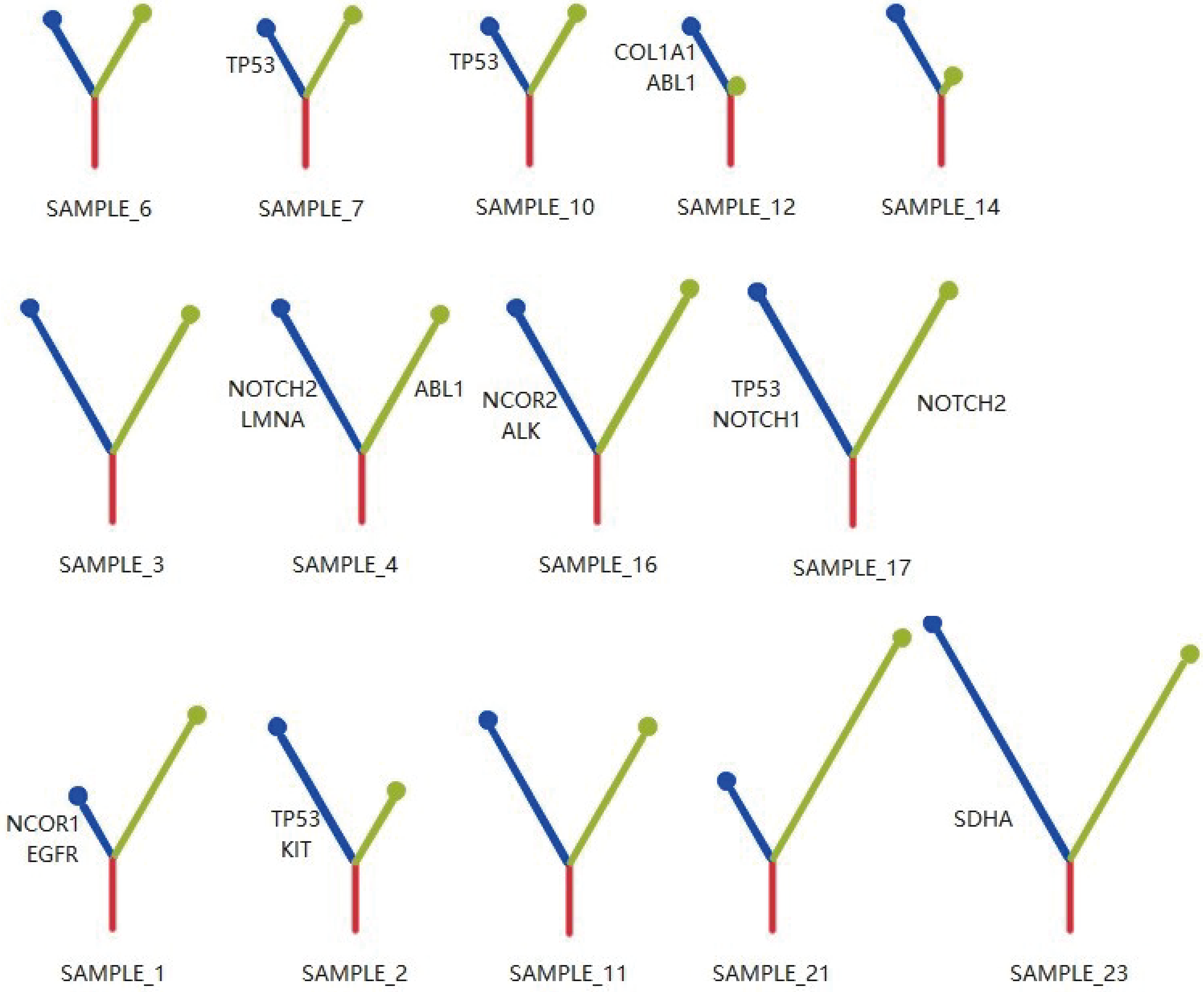
TP53
TP53
COL1A1
ABL1
SAMPLE_6
SAMPLE_7
SAMPLE_10
SAMPLE_12
SAMPLE_14
NOTCH2
LMNA
ABL1
NCOR2
ALK
TP53
NOTCH1
NOTCH2
SAMPLE_3
SAMPLE_4
SAMPLE_16
SAMPLE_17
NCOR1
EGFR
TP53
KIT
SDHA
SAMPLE_1
SAMPLE_2
SAMPLE_11
SAMPLE_21
SAMPLE_23
