# Supplementary figures and images for: Whole-Exome Sequencing Reveals the Genomic Features of the Micropapillary Component in Ground-Glass Opacities
Source: Cancers (Basel). 2022 Aug 27;14(17):4165. doi: 10.3390/cancers14174165 (PMC9454937; doi:10.3390/cancers14174165)

**A**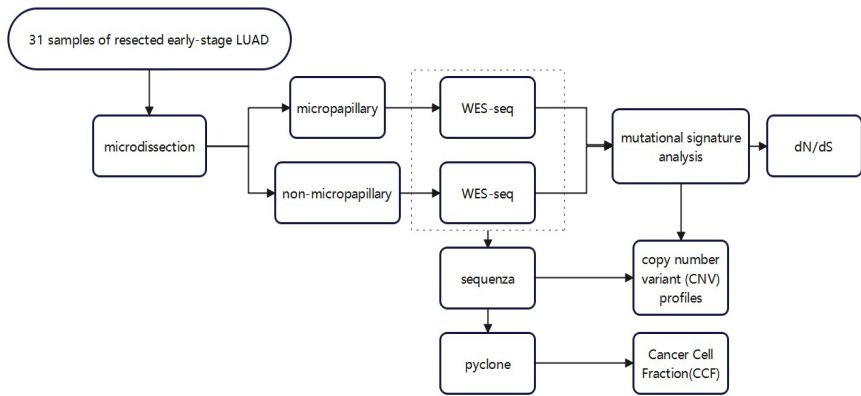**B**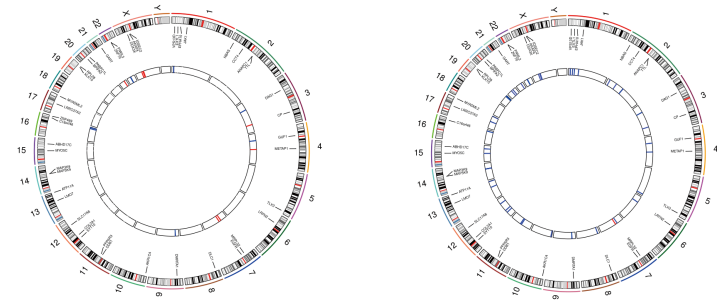**C**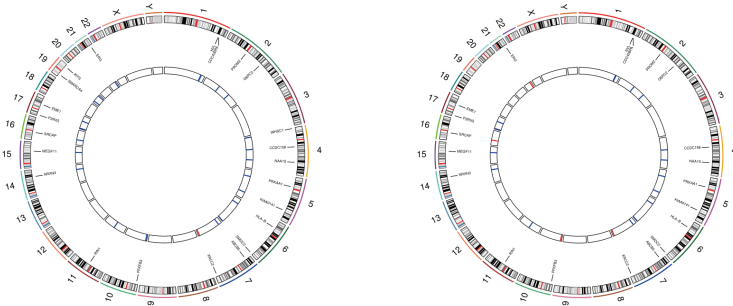**D**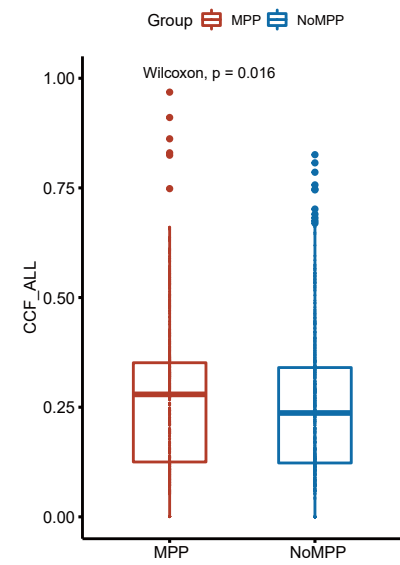**E**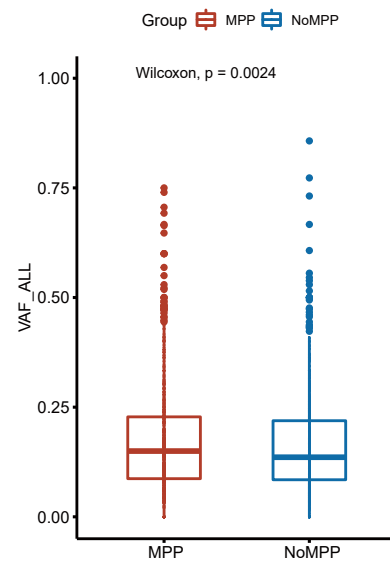**F**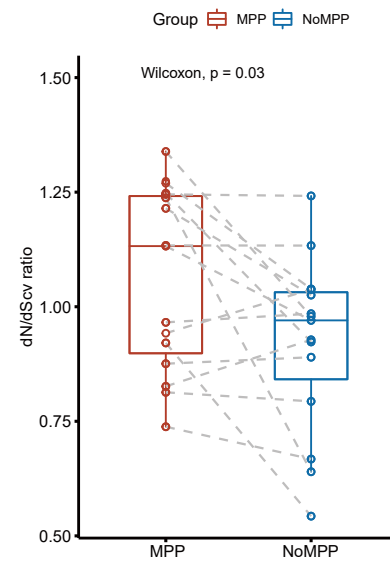

Supplement: Supplementary file 1 [file cancers-14-04165-s001.zip › cancers-1855002-supplementary-for xml/Fig.S1.pdf]
